# Supplementary material for: Research on the key technology of high-precision waveform synchronization identification and measurement
Source: PLoS One. 2024 Mar 5;19(3):e0299027. doi: 10.1371/journal.pone.0299027 (PMC10914255; doi:10.1371/journal.pone.0299027)
Supplement: Attachment — Submitted filename: Review comments.docx [file pone.0299027.s002.docx]

I have carefully read the manuscript entitled “Research on the key technology of high precision waveform synchronization identification and measurement” for possible publication in PLoS One.

The authors developed an amplifying and attenuating preselector circuit to measure waveforms across various values. Afterward, the authors adopted high-precision analog-to-digital conversion and a fast Fourier algorithm to capture and analyze the critical parameters of the waveforms. The combination of hardware and software was an excellent idea. Simulation and experimental data demonstrate the feasibility and efficiency of the proposed circuit and fast recognition algorithm. This work will be of interest to the waveform recognition community, especially those applications with high precision and quick response requirements. A minor revision has to be done before this manuscript can be accepted for the public in PLoS One.

1. In the introduction section, waveform identification and parameter measurement are crucial for signal processing with a wide range of applications, and the authors need to add relevant literature.
2. Fig 1. The block diagram of the system structure. Resize modules for better graphics, such as putting the letters "External" on the same line.
3. Limitation discussion. The proposed method has significant effects on essential waveform judgment and calculation speed. However, whether the input signals with irregular or non-periodic can also be measured accurately and quickly could be given additional clarification.
4. Pay attention to expression; the current manuscript needs to be written concisely and straightforwardly.
