# Supplementary material for: Research on the key technology of high-precision waveform synchronization identification and measurement
Source: PLoS One. 2024 Mar 5;19(3):e0299027. doi: 10.1371/journal.pone.0299027 (PMC10914255; doi:10.1371/journal.pone.0299027)
Supplement: Attachment — Submitted filename: Response to Reviewers-20240130.docx [file pone.0299027.s003.docx]

Dear editor,

Thanks a lot for the message to our manuscript (ID: PONE-D-23-24833), and thank the reviewers for their valuable comments. In the revised manuscript all reviewers’ comments have been addressed. Please find a point-by-point response to the reviewers’ comments in the following pages.

We hope our manuscript could be accepted by the prestigious *PLOS ONE.*

Best regards,

Dan

------------------------------------------------------------------

Dan Gui, Ph D, Associate Professor

Institute of Electronic Engineering

Wuhan Vocational College of Software and Engineering

Guanggu Avenue 117, Wuhan 430205, China

E-mail: 71230412@qq.com

------------------------------------------------------------------

**Response to reviewers’ comments**

**Reviewer #1**

I have carefully read the manuscript entitled “Research on the key technology of high precision waveform synchronization identification and measurement” for possible publication in PLoS One.

The authors developed an amplifying and attenuating preselector circuit to measure waveforms across various values. Afterward, the authors adopted high-precision analog-to-digital conversion and a fast Fourier algorithm to capture and analyze the critical parameters of the waveforms. The combination of hardware and software was an excellent idea. Simulation and experimental data demonstrate the feasibility and efficiency of the proposed circuit and fast recognition algorithm. This work will be of interest to the waveform recognition community, especially those applications with high precision and quick response requirements. A minor revision has to be done before this manuscript can be accepted for the public in PLoS One.

**Reply:** Many thanks for the kind words.

**Major comment-1:** In the introduction section, waveform identification and parameter measurement are crucial for signal processing with a wide range of applications, and the authors need to add relevant literature.

**Reply:** The relevant literature on the wide range of applications of waveform identification and parameter measurement has been added to the revised manuscript to illustrate its important role in the field of signal processing.

**Major comment-2:** Fig 1. The block diagram of the system structure. Resize modules for better graphics, such as putting the letters "External" on the same line.

**Reply:** Thank you so much for your careful check, the overall structure of Figure 1 has been optimized in the revised manuscript.

**Major comment-3:** Limitation discussion. The proposed method has significant effects on essential waveform judgment and calculation speed. However, whether the input signals with irregular or non-periodic can also be measured accurately and quickly could be given additional clarification.

**Reply:** Thank you very much for your constructive comments. The limitation discussion has been added to the Conclusion and Discussion section of the revised manuscript. For irregular or non-periodic input signals, the method proposed in the manuscript may be reduced in measurement accuracy and response speed due to the limited signal judgment method and insufficiently high chip sampling frequency. The replacement of higher performance chip and smarter recognition method case can be effectively improved，which is the next part of our upcoming research.

**Major comment-4:** Pay attention to expression; the current manuscript needs to be written concisely and straightforwardly.

**Reply:** Language and content have been refined and revised throughout the text in the revised manuscript.

**Reviewer #2**

Waveform synchronization and measurement is an important branch of instrumentation, while the current manuscript has not presented sufficient proof to reach the publication threshold of Plos One. Following is some concerns:

**Major comment-1**：Frequency error of 0.1% is rather far from an advanced progress, although it depends on measurement time, SNR, etc. To what extend one can push the precision and accuracy, formulus 2 in a RSI paper (doi: 10.1063/5.0002377) has already clarified.

**Reply:** Thank you for your comments. For localized fine frequency measurement, the lock-in frequency measurement method in the paper (doi: 10.1063/5.0002377) has high accuracy and efficiency. But for wide frequency ranges, the FFT-like methods are general, and considering the cost, application range and other factors, the FFT-based measurement methods in this manuscript can meet the requirements of waveform measurement in both the fundamental frequency range and wider frequency ranges.

**Major comment-2**：Comparing to a lock-in amplifier, mV-range measurement is far from scientific researches, so authors are strongly recommended to upgrade the measurement construction for high precision measurement with lower cost.

**Reply:** Thanks to these suggestions, this manuscript is aimed at generalized scenarios to achieve arbitrary waveform identification and key parameter measurement. in the next step, we will upgrade the measurement structure with specific applications on low cost and high accuracy.

**Major comment-3**：STM32F4 is relatively slow for electronic feedback, so it is only suitable for some situations where delay of seconds is acceptable. Nevertheless, authors have not clearly shown a necessary application.

**Reply:** Considering the cost, universalization and other factors, STM32F4 is a widely used microcontroller that can meet the response speed requirements for waveform recognition and parameter measurement in basic and wider frequency ranges. Though upgrading the microprocessor chip, faster response speed can be realized.
